# Nutritional issues and considerations in the elderly: an update

**DOI:** 10.3325/cmj.2020.61.180

**Published:** 2020-04

**Authors:** Darija Vranešić Bender, Željko Krznarić

**Affiliations:** 1 Unit of Clinical Nutrition, Department of Internal Medicine, University Hospital Centre Zagreb, Zagreb, Croatia *dvranesi@kbc-zagreb.hr*; 2 University of Zagreb School of Medicine, Zagreb, Croatia

Nutritional aspects of some common clinical conditions of older age are often neglected. Therefore, the aim of this essay is to describe the prevalence, recognition, and general management of four common and interrelated clinical problems in the elderly: sarcopenia, dysphagia, chronic wounds, and dementia. In the modern era of clinical nutrition tailored to elderly patients, we should encourage a multidimensional personalized approach that includes assessment, counseling, dietary modification, targeted oral nutritional supplements, adjusted physical activity or therapy, and psychosocial support (1,2).

## Sarcopenia and frailty

European Working Group on Sarcopenia in Older People (EGWGSOP) defined sarcopenia as loss of skeletal muscle mass together with low muscle strength and inadequate physical performance (3). Sarcopenia prevalence in the elderly in rehabilitation centers ranges up to 50%. Furthermore, 15% hospitalized non-sarcopenic patients upon admission develop sarcopenia during the hospital stay (4). Primary sarcopenia is associated with aging (muscle mass atrophy) and secondary sarcopenia with underlying diseases, lack of physical activity, or inadequate protein intake (5). Sarcopenic patients have a greater risk of a prolonged hospital stay, delayed healing, wound infections, and poor surgery outcomes (6). Owing to aging effect on bone and muscle, sarcopenia is often associated with osteoporosis, and both cause frailty in the elderly. Frailty is considered to be a multidimensional geriatric syndrome as it involves severe deterioration of bodily systems and functions, and its pathogenesis involves both physical and social dimensions (7). Sarcopenia, as well as frailty, is associated with a number of negative consequences, such as increased risk of falls, disability, complications of various chronic diseases, greater need for health care and medication, increased mortality, and poorer quality of life.

Sarcopenic obesity is characterized by decreased lean body mass together with excess fat mass. Obesity aggravates sarcopenia, impairs physical function, and increases mortality rates (7). Elderly patients are more often sarcopenic and obese, especially if they have comorbidities such as cardiovascular disease, diabetes mellitus type 2, chronic obstructive pulmonary disease, and osteoarthritis, and a lack of physical activity.

The definition of sarcopenia encompasses low values for the combination of the following parameters: 1) muscle strength, 2) muscle quality or quantity, and 3) physical performance. Several techniques are used for the measurement of muscle mass (eg, bioimpedance analysis, anthropometric measurements, computed tomography, magnetic resonance imaging, and dual energy x-ray absorptiometry) and muscle function and physical performance (eg, handgrip strength, Short Physical Performance Battery, gait speed test) (8). New guidelines (EWGSOP2) suggest that the first diagnostic criterion for sarcopenia is low muscle strength, which can be easily established with dynamometry (9). EWGSOP2 defines low handgrip strength as <16 kg for women and <27 kg for men (7). Furthermore, the same working group described subcategories of sarcopenia as chronic (duration 6 or more months) and acute (duration less than 6 months).

There is still no specific pharmacologic intervention for sarcopenia, making conservative measures the mainstay of the management. These measures include resistance training together with nutrition support – supplementation with protein (1–1.5 g/kg/d), specific amino acids (leucine and its metabolite β-hydroxy-β-methylbutyrate), and vitamin D (800-1000 IU/d) (10-14). In sarcopenic obese patients, we recommend energy restricted diet under medical supervision, together with supplementation with the key nutrients, an approach that is even more challenging than the approach used to treat malnutrition related sarcopenia.

## Dysphagia, presbyphagia, and sarcopenic dysphagia

Swallowing physiology changes with advancing age, therefore, dysphagia (swallowing difficulty) is recognized as a health problem of the elderly population. Dysphagia is diagnosed in as many as 40% of people in long-term care, and in 50%-75% of nursing homes residents (15). The risk factors for dysphagia in this population are age-related changes in swallowing physiology and common diseases of older age (16). Presbyphagia is a term used to describe swallowing changes and difficulties in otherwise healthy older adults (8). Sarcopenic dysphagia develops as a result of muscle mass and function loss in skeletal and swallowing muscles (4). Dysphagia is an independent factor of malnutrition, with a high prevalence in patients with neurodegenerative diseases (29%-64% in stroke and over 80% in dementia) (17).

The individual approach of a multidisciplinary team, especially collaboration between speech therapists, physicians, and nutritionists, is extremely important in patients with dysphagia to ensure timely recognition and adequate nutritional support. Clinical nutrition, which entails different modalities of treatment, from diet therapy to specialized artificial nutritional support, is today an indispensable segment of treatment for patients with dysphagia, especially patients with advanced chronic diseases. Changes in the consistency of liquids and foods, use of enteral formulas with adjusted texture, or the administration of food and liquid thickening preparations may facilitate the ingestion process and reduce the risk of aspiration. Nutritional support for people with dysphagia may improve the quality of life and prevent the negative impact of malnutrition on the course and outcome of treatment (18).

## Chronic wounds and pressure ulcers

Frailty and sarcopenia in the elderly often occur together with chronic wounds and pressure ulcers in malnourished elderly patients. The prevalence of decubital ulcers in geriatric long-term care centers has reached significant proportions worldwide, with up to 30% of patients admitted to institutions with pre-existing decubitus (19). Not all wounds are equal: pressure ulcer is different from diabetic foot ulcer or a burn. However, when it comes to nutrition, similar nutritional rules are applied for different kind of wounds (20).

The process of wound healing depends on a complex cascade of physiologic and immunologic processes, as well carefully designed nutritional support. Although very often overlooked, medical nutrition therapy is an important part of the prevention and therapy of chronic wounds and pressure ulcers. Therefore, nutritional care must be tailored to each individual patient. The recommended energy intake of malnourished geriatric patients with chronic wounds or decubitus ranges from 30 to 40 kcal/kg/d, while protein intake should be adjusted to the range of 1.2-1.5 g/kg/d (21). Some specific nutrients that are involved in the wound healing process should be considered as pharmaconutrients, and those are mainly proteins, arginine, glutamine, zinc, β-hydroxy-β-methylbutyrate, and vitamin C (22). The effect of these nutrients depends on the local circulation in the wound region, which determines efficient nutrient transfer and local metabolism as well as the elimination of toxic cellular metabolites (23).

## Dementia

Demented elderly patients frequently suffer from malnutrition, involuntary weight loss, and dehydration, which may appear at any stage of the disease (24). Malnutrition in dementia can be caused by a variety of factors, such as anorexia resulting from polypragmasy, inadequate oral intake (patients forget to eat), depression, feeding apraxia, or less frequently increased energy needs due to hyperactivity. Dementia and malnutrition form a vicious circle because dementia *per se* affects patient’s ability and desire to eat and drink. Impaired nutritional status can increase the risk of mortality and morbidity and can impair the quality of life and clinical outcomes (25).

Patients with dementia should undergo nutritional risk screening in order to detect those who can benefit from early nutritional intervention. In the situations when additional energy or macronutrients are needed food fortification with energy and protein together with behavioral modifications is recommended (26). Such dietary manipulation can be achieved using high-energy (high quality plant oils, nuts, seeds, honey) and high-protein (dairy, egg-white, legumes, meat, and fish) foods as well as commercial modular preparations consisting of one type of macronutrient (carbohydrate, protein, or fat) in powder or liquid form. Most of these preparations have no taste and can be mixed in foods such as milk, yogurt, sauces, or soups. Several studies have shown that this approach can increase energy and protein intake. Frequent in-between meals are practical and effective aid for the nutritional support. The little so-called finger-food meals are also very well accepted by demented people (27).

Patients with mild cognitive impairment (MCI) might benefit from interventions with special mixture of nutrients, cofactors, and precursors. Dietary supplements consisting of a single nutrient did not show significant cognitive benefits in patients with MCI, but a more complex, synergistic formulation of nutrients should be taken into consideration. Oral nutritional supplements containing long-chain omega-3 fatty acids, B vitamins, vitamin C, vitamin E, nucleotide uridylate, choline, and selenium are proven to contribute to normal function of neuronal membranes and synapses (28). Particular attention has been paid to the substitution of folic acid, vitamin B6, and vitamin B12, which synergistically reduce homocysteine, a marker whose elevated levels correlate with an increased risk of dementia and cognitive impairment. However, existing data on improvement in cognitive functions by B-vitamin supplementation and lowering homocysteine levels are still conflicting (29).

Patients with advanced severity of dementia may develop dysphagia, which may be an indication for enteral feeding. ESPEN guidelines on nutrition in dementia suggest that artificial nutrition should be used in patients with moderate or mild dementia during the periods of insufficient oral intake caused by conditions that are potentially reversible. In patients with severe dementia or those in the terminal phase who are immobile, non-communicable, or completely dependent, artificial nutrition is not indicated (27). Although dementia is one of the indications for artificial feeding via percutaneous endoscopic gastrostomy (PEG), the procedure may also be connected with increased short-term mortality after the placement. Therefore, PEG should be performed in carefully selected patients, and the risks and benefits ratio for each clinical case should be assessed with respect to general prognosis and patients' preferences (27,30).

## Conclusion

Nutritional aspects of common clinical conditions presenting with decline of nutritional status in the elderly require raising awareness in broad medical society. Timely recognition and adequate nutrition support may prevent or alleviate consequences of malnutrition and reduce morbidity and mortality in elderly populations.
